# Medullary nephrocalcinosis, distal renal tubular acidosis and polycythaemia in a patient with nephrotic syndrome

**DOI:** 10.1186/1471-2369-13-66

**Published:** 2012-07-26

**Authors:** Suneth Karunarathne, Yapa Udayakumara, Dumitha Govindapala, Harshini Fernando

**Affiliations:** 1 National hospital of Sri Lanka, Regent street, Colombo 8, Sri Lanka

**Keywords:** Medullary nephrocalcinosis, Nephrotic syndrome, Polycythaemia, Renal tubular acidosis type I

## Abstract

**Background:**

Medullary nephrocalcinosis and distal renal tubular acidosis are closely associated and each can lead to the other. These clinical entities are rare in patients with nephrotic syndrome and polycythaemia is an unusual finding in such patients. We describe the presence of medullary nephrocalcinosis, distal renal tubular acidosis and polycythaemia in a patient with nephrotic syndrome due to minimal change disease. Proposed mechanisms of polycythaemia in patients with nephrotic syndrome and distal renal tubular acidosis include, increased erythropoietin production and secretion of interleukin 8 which in turn stimulate erythropoiesis.

**Case presentation:**

A 22 year old Sri Lankan Sinhala male with nephrotic syndrome due to minimal change disease was investigated for incidentally detected polycythaemia. Investigations revealed the presence of renal tubular acidosis type I and medullary nephrocalcinosis. Despite extensive investigation, a definite cause for polycythaemia was not found in this patient. Treatment with potassium and bicarbonate supplementation with potassium citrate led to correction of acidosis thereby avoiding the progression of nephrocalcinosis and harmful effects of chronic acidosis.

**Conclusion:**

The constellation of clinical and biochemical findings in this patient is unique but the pathogenesis of erythrocytosis is not clearly explained. The proposed mechanisms for erythrocytosis in other patients with proteinuria include increased erythropoietin secretion due to renal hypoxia and increased secretion of interleukin 8 from the kidney. This case illustrates that there may exist hitherto unknown connections between tubular and glomerular dysfunction in patients with nephrotic syndrome.

## Background

Childhood nephrotic syndrome with bland urinary sediment is usually managed as minimal change disease and renal biopsy is not necessary for the diagnosis unless there are unusual features [1]. Although some children achieve a sustained remission or reduced frequency of relapses as they grow older, others tend to have repeated relapses during young adulthood.

Distal renal tubular acidosis is due to defective proton secretion from the alpha (α) intercalated cells of the distal tubule caused by dysfunction of the H^+^/K^+^ antiporter on the apical membrane [2]. This leads to failure of H^+^ excretion thereby causing systemic acidosis and potassium depletion. Inability to lower the urine pH below 5.3 in the presence of systemic acidosis is the diagnostic hallmark of type I or distal renal tubular acidosis.

Causes of distal renal tubular acidosis include autoimmune diseases such as Sjögren's syndrome, systemic lupus erythematosus and rheumatoid arthritis. Hereditary distal tubular acidosis can occur due to mutations of bicarbonate transporter called “band 3” of the basolateral membranes of the intercalated cell which could be either autosomal dominant (Western Europe) or recessive (south east Asia) [3]. Cirrhosis, nephrocalcinosis, renal transplantation, sickle cell anaemia, and obstructive uropathy are other known aetiological factors. Drug induced renal tubular acidosis can occur due to zoledronate, ifosfamide, amphotericin B and lithium carbonate.

Hypercalciuria, hypocitraturia and elevated urinary pH observed in distal renal tubular acidosis can lead to nephrocalcinosis and may cause renal calculi, obstructive uropathy and renal failure necessitating surgical or endoscopic stone extraction [4].

Medullary nephrocalcinosis is the diffuse calcification of the renal medulla due to deposition of calcium salts within the parenchyma. Minute calcifications seen in early stages may not be visible, but in later stages extensive calcifications become visible in plain x ray films and at times will give rise to renal colic and hydronephrosis when dislodged calcific foci travel down the ureters.

Medullary sponge kidney, a developmental disorder causing dilatation of the collecting ducts may independently cause both nephrocalcinosis and distal renal tubular acidosis with associated metabolic abnormalities. This condition is characterized by diffuse dilatation of precalyceal collecting ducts [5]. Associated biochemical abnormalities include hypercalciuria, hypocitraturia and defective urinary acidification giving rise to distal renal tubular acidosis, nephrocalcinosis and increased risk or urinary tract infections.

We describe a patient with nephrotic syndrome who is currently in remission with steroid sparing immunomodulator therapy who was found to have idiopathic erythocytosis. Upon further investigation he was having medullary nephrocalcinosis ultrasonically and type I renal tubular acidosis.

Proposed mechanisms for erythrocytosis in patients with renal tubular acidosis and nephrotic syndrome include increased erythropoietin secretion due to intra renal hypoxia and increased secretion of interleukin 8 which in turn stimulate erythropoiesis. These mechanisms are yet to be confirmed biochemically [6].

## Case presentation

A 22 year old Sri Lankan Sinhala male who was being treated for steroid sensitive childhood nephrotic syndrome attended for routine clinic follow up.

There were no relapses for last two years and he did not complain of frothuria or ankle oedema. His routine medication was continued and he was reviewed with a urine full report and full blood count as he was plethoric.

The patient was diagnosed as having nephrotic syndrome at the age of two years and had responded to steroids well. Despite good steroid response he continued to have frequent relapses as the follow up visits were not regular due to family’s socioeconomic problems.

Renal biopsy was not performed earlier because of the good steroid response and absence of active sediment in urine. I.e. managed as minimal change disease. At the age of twenty one years he was found to have steroid induced osteoporosis, short stature and acne. He was started on alendronate, vitamin D and calcium supplements. Oral mycophenolate mofetil 250 mg twice a day was started as a steroid sparing agent one year previously.

Full blood count revealed that he had polycythaemia (Haemoglobin concentration of 17.8 g/L with a haematocrit of 54%) repeated full blood counts confirmed the presence of polycythaemia. Urine full report was negative for protein and red cells at this stage. An array of investigations were performed to search for a cause of the polycythaemia after the history excluded active or passive smoking and the physical examination did not show any murmurs suggestive of intra cardiac shunts or intra abdominal masses. There was no evidence to suggest a chronic lung disease on physical examination of the respiratory system.

A transthoracic echocardiogram and a transoesophageal echocardiogram did not reveal any shunts or evidence of pulmonary hypertension. Arterial blood gas analysis showed a metabolic acidosis but there was no hypoxia. (PH-7.3, PCO_2_-31 mmHg, PO_2_-92 mmHg, HCO_3_^-^ -15.3 mmol/l) Serum erythropoietin level was normal at 6.2mIU/ml (3.7-29.5). Since there were no secondary causes such as renal failure or diabetic ketoacidosis which could explain the metabolic acidosis, renal tubular acidosis was suspected. An acid loading test was performed (Table 1) which showed failure of urinary acidification in spite of systemic acidosis.

**Table 1:** Acid loading test

	**09.30 hrs (immediately prior to acid loading)**	**At 15.30 hrs**
Urine pH	6.91	6.86
Arterial PH	7.42	7.29
PCO2	34	21
HCO3	22	10.1

Results demonstrate the failure of urine to acidify in the presence of systemic acidosis. Suggestive of distal renal tubular acidosis.

Urinary anion gap was positive favoring a diagnosis of Distal or type I renal tubular acidosis (Table 2) Serum potassium level remained persistently low. Patient was diagnosed as having a hypokalaemic, hyperchloraemic metabolic acidosis, most probably distal renal tubular acidosis (RTA). On further investigation, he was found to have medullary nephrocalcinosis ultrasonically but abdominal X ray did not show any calcifications. Other relevant investigation findings were as follows: serum calcium 2.6 mmol/l (2.1-2.55),serum magnesium 0.7 mmol/l(0.75-1.2),serum Phosphate 1.4 mmol/l (0.8-1.5), urine Calcium/Creatinine ratio of a fasting urine sample 0.7 (<0.4), Intact PTH 17 pg/ml(10–65) ALP 201 IU/l, Free T4 1.39 ng/dl(0.7-1.5), TSH 1.27uIU/ml(0.3-2.0), 24 Hr urine protein excretion 0.6 g, Creatinine clearance 94 ml/min.

**Table 2:** Serum electrolytes, blood and urine pH and blood and urine anion gap

**Parameter**	**Value meq/l**
Serum Na^+^	138
Serum K^+^	2.4
Serum Cl^-^	114
Urine Na^+^	60
Urine K^+^	19.5
Urine Cl^-^	78
Blood Anion gap	9.1
Urine Anion gap	2
Blood PH	7.33
Urine PH	7.34

Bone marrow aspiration biopsy was compatible with idiopathic erythrocytosis as JAK-2 V617F and Exon 12 mutations were negative.

The final diagnosis was steroid sensitive nephrotic syndrome, medullary nephrocalcinosis, type 1 distal renal tubular acidosis and idiopathic erythrocytosis. He was prescribed potassium citrate oral solution(potassium citrate monohydrate 22 g/dl) two tablespoons twice a day. He was also prescribed oral atorvastatin and alendronate. An arterial blood gas analysis one month after commencing therapy showed correction of the acidosis with normal bicarbonate level. ABG after treatment was as follows: PH- 7.40, PCO_2_ -39 mmHg, HCO_3_^–^ 24 mmol/l.

Since his follow up haematocrit was 52% venesection was not performed. His renal functions and creatinine clearance continued to be normal. Serum potassium level was normalized at 4.2 mmol/l. Although his biochemical abnormalities were thus corrected with therapy he was advised to continue clinic follow up to detect progression of nephrocalcinosis, renal impairment, relapses of nephrotic syndrome and monitoring of haematocrit to institute venesection if and when appropriate.

## Discussion

Although the association of medullary nephrocalcinosis and type I hypokalaemic renal tubular acidosis is well recognized [7,8] the association of these two clinical entities with nephrotic syndrome and essential thrombocytosis is rare.

Association of medullary nephrocalcinosis, distal renal tubular acidosis and erythrocytosis is reported in a patient with nephrotic syndrome due to primary FSGS. In this patient the proposed mechanisms for erythrocytosis were increased erythropoietin secretion due to intrarenal hypoxia and increased secretion of interleukin 8 which in turn can stimulate bone marrow [6].

Balogun RA et al. describes medullary nephrocalcinosis, distal renal tubular acidosis and proteinuria in a patient with focal segmental glomerulosclerosis [9] but there was no polycythaemia. Another report by Agroyannis et al. describes a patient with medullary nephrocalcinosis, type I distal renal tubular acidosis and erythocytosis [10] but this patient did not have nephrotic syndrome or proteinuria.

The association of minimal change disease, proteinuria and erythrocytosis were observed in four previous reports [11-14]. But nephrocalcinosis or renal tubular acidosis were not described in these patients. Despite the implication of increased erythropoietin levels in the pathogenesis of erythrocytosis, erythropoietin level was found to be normal in two of these reports while the erythropoietin levels were not measured in other two.

The thrombocytosis was not due to renal overproduction of erythropoietin as the erythropoietin level was normal in our patient. Therefore the other proposed mechanism of erythocytosis i.e. increased renal production of interleukin 8 has to be considered in this patient but the measurement of interleukin levels was not done in our patient due to resource constraints.

Treatment with potassium citrate to supplement potassium and bicarbonate normalized the arterial pH, bicarbonate level and potassium level. It is expected that this treatment will prevent the development of renal failure due to progression of nephrocalcinosis while continuation of mycophenolate mofetil will help to maintain the remission of nephrotic syndrome. The management of essential thrombocytosis will depend on the packed cell volume and the symptoms during follow up visits.

If and when the patient develops further relapses of nephrotic syndrome a renal biopsy has to be considered to understand the pathology of the glomerular lesion so as to direct therapy.

## Conclusion

Distal renal tubular acidosis, nephrocalcinosis and polycythaemia occurring in a patient with nephrotic syndrome is rare. Increased erythropoietin levels due to intrarenal hypoxia and IL 8 mediated bone marrow stimulation are the proposed mechanisms of erythrocytosis in patients with nephrotic syndrome. Patients with distal renal tubular acidosis should be treated preferentially with potassium citrate.

### Consent

Written informed consent was obtained from the patient for publication of this Case report and any accompanying images. A copy of the written consent is available for review by the Series Editor of this journal.

## Competing interests

The authors declare that they have no competing interests

## Authors’ contributions

All four authors were involved in management of the patient. SK prepared the manuscript. YU and DG provided input during manuscript preparation and literature survey. HF critically appraised the manuscript and edited the scientific data. All authors read and approved the final manuscript.

## Authors’ information

SK is a postgraduate trainee in general medicine at National hospital of Sri Lanka, which is the final tertiary care referral centre in Sri Lanka. YU and DG are senior registrars in medicine at the same institute. HF is a consultant physician at the National hospital of Sri Lanka.

## Pre-publication history

The pre-publication history for this paper can be accessed here:

http://www.biomedcentral.com/1471-2369/13/66/prepub
